# The immunization-induced antibody response to the *Anaplasma marginale* major surface protein 2 and its association with protective immunity

**DOI:** 10.1016/j.vaccine.2010.02.067

**Published:** 2010-05-07

**Authors:** Susan M. Noh, Yan Zhuang, James E. Futse, Wendy C. Brown, Kelly A. Brayton, Guy H. Palmer

**Affiliations:** a Animal Disease Research Unit, Agricultural Research Service, U.S. Department of Agriculture, Pullman, WA 99164-7030, United States; b Program in Vector-Borne Diseases, Department of Veterinary Microbiology and Pathology, and School for Global Animal Health, Washington State University, Pullman, WA 99164-7040, United States

**Keywords:** *Anaplasma marginale*, Antibody, Outer membrane proteins

## Abstract

Many vector-borne pathogens evade clearance via rapid variation in their immunogenic surface expressed proteins. This is exemplified by *Anaplasma marginale*, a tick-borne bacterial pathogen that generates major surface protein 2 (Msp2) variants to provide for immune escape and allow long-term pathogen persistence. In contrast to persistence following infection, immunization with a surface protein complex, which includes Msp2, induces a response that prevents infection upon challenge. We hypothesized that the immune response induced by immunization altered the anti-Msp2 antibody repertoire as compared to that induced during infection, shifting the immune response toward conserved and thus broadly protective epitopes. The antibody response to the conserved (CR) and hypervariable (HVR) regions encoded by the full set of *msp2* variant alleles was determined for immunized animals prior to challenge and non-immunized, infected animals. While both groups of animals had a similar antibody repertoire in terms of breath and magnitude, the titers to the Msp2 CR were strongly correlated (*p* < 0.005) with control of bacteremia only in the infected animals. Among the immunized animals, there was no correlation between the breadth or magnitude of the anti-Msp2 antibody response and either complete protection from infection or control of bacteremia. This is consistent with separate immunologic mechanisms being responsible for control of bacteremia in infected animals as compared to immunized animals and suggests that conserved outer membrane proteins other than Msp2 are responsible for the complete clearance observed following challenge of vaccinees.

## 1 Introduction

**Fig. 1:**
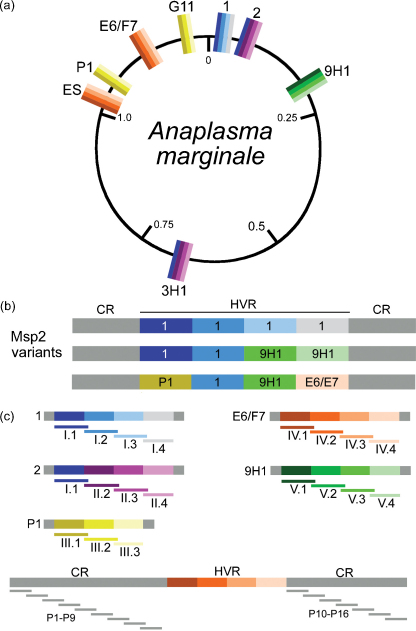
The circular genome of the St. Maries strain of *A. marginale* (a) has one Msp2 expression site (ES), and five unique Msp2 pseudogenes which serve as donors for antigenic variation. Two of the Msp2 pseudogenes are duplicated (P1 and G11, 2 and 3H1) as indicated by matching colors. A linear depiction of the Msp2 expression site illustrates the source of variability within the Msp2 expression site (b). 30-mer peptides representing the CR and all possible pseudogene segments (c) were constructed and used to map the anti-Msp2 antibody response in this study.

Infection with many vector-borne pathogens including *Theileria* spp., *Anaplasma* spp., *Babesia* spp., *Borrelia* spp., and *Plasmodium* spp. results in long-term persistent infection due to the pathogen's ability to evade the host immune response. This ability is in large part due to generation of outer membrane protein antigenic variants. For example, infection with *Anaplasma marginale*, a bacterial pathogen of cattle, generally results in life-long persistence in the mammalian host. Persistence is attributed primarily to rapid shifts in the surface coat structure and specifically variation in the highly immunogenic major surface protein 2 (Msp2). The expressed copy of Msp2 is composed of a central hypervariable region that is flanked by highly conserved regions (Fig. 1a and b). The variation is generated by gene conversion in which one of multiple *msp2* donor alleles is recombined into a single, operon-linked expression site [1], [2], [3]. The donor alleles have 5′ and 3′ regions which are identical to the expression site copy and flank a unique allele-specific hypervariable domain [1], [4]. These donor alleles are termed functional pseudogenes as their 5′ and 3′ regions are truncated, they lack the function elements for *in situ* transcription, and are only expressed following recombination into the single expression site [1], [4].

During infection, Msp2 represents dominant antigens recognized by sera from cattle infected with *A. marginale*. The anti-Msp2 specific antibody response is predominantly directed toward the hypervariable region rather than the flanking conserved regions [5], [6]. However, the hypervariable region of newly emergent variants is not recognized by existing antibody [7], [8]. Thus, generation of Msp2 variants allows for immune escape and long-term pathogen persistence [8], [9]. In contrast to infection, where clearance does not occur, immunization with either purified *A. marginale* outer membranes or cross-linked outer membrane protein complexes induces complete protection against infection in 40–70% of vaccinees, and protection against anemia and high-level bacteremia in nearly all animals [7], [10], [11]. Protection correlates with high IgG antibody titers against surface-exposed polypeptides, including Msp2 [7]. While protection associates with the IgG response to outer membrane proteins, the specific epitope targets and characteristics of this protective immune response remain unknown. In the experiments reported here we investigated the breadth and magnitude of the anti-Msp2 antibody response associated with the control of bacteremia in infection, and in the prevention of infection and control of bacteremia in immunized animals.

The first goal of these experiments was to determine if immunization altered the magnitude or epitope specificity of the anti-Msp2 responses as compared to infection; specifically whether immunization as compared to infection shifted the antibody response, in terms of the breadth or magnitude, toward the conserved regions of Msp2. This immunity against conserved region epitopes could prevent immune escape of new variants and result in the clearance observed following challenge of immunized animals but not during natural or experimental infection. The second goal of these experiments was to determine if the breadth or magnitude of the anti-Msp2 antibody response correlated with control of bacteremia in infected animals or prevention or control of bacteremia in immunized animals. To address these questions, animals were immunized with purified outer membranes or cross-linked surface proteins from the St. Maries strain of *A. marginale*, and the resulting specific antibody responses to the hypervariable (HVR) and conserved (CR) regions of Msp2 were mapped and titered. Vaccinees were then challenged with the homologous strain of *A. marginale*. Importantly, the St. Maries strain, for which the complete genome sequence is available, was used in these experiments, thus allowing mapping of the Msp2 expressed variants to their original donor pseudogene alleles, analysis of all possible combinations of the HVR, and comprehensive testing of the epitope specificity induced by immunization versus infection.

## 2 Methods

### 2.1 Immunization and challenge

The immunization and challenge have been previously reported in detail [11]. Briefly, two groups of five calves each were immunized 5 times at 3-week intervals with approximately 35 μg of either *A. marginale* outer membranes or protein complexes suspended in 1 mg of saponin in a total volume of 1 ml administered subcutaneously. The third group of five calves was similarly immunized on the same schedule using only adjuvant. Four months after the last immunization, all calves were challenged intravenously with approximately 1 × 10^4^ *A. marginale* (St. Maries strain) in 1 ml Hank's balanced salt solution. Starting 10 days post-challenge, the packed cell volume and bacteremia, as defined by the percent of infected erythrocytes, were determined daily for all the animals.

### 2.2 PCR to confirm infection status

PCR was used to confirm the lack of infection in the four challenged vaccinees that did not develop microscopically detectable bacteremia based on daily blood smear examination. DNA was isolated from whole blood using a Puregene DNA isolation kit (Qiagen, Valencia CA). Primers that specifically amplify *msp5*, a single copy gene, were used to detect *A. marginale*, as previously described in detail [12], [13]. Amplification was performed in 50 μl volume with 35 cycles of melting at 94 °C for 15 s, annealing at 65 °C for 58 s, and extension for 71 s at 72 °C.

### 2.3 Measurement of segment specific antibodies

All conserved (Table 1) and hypervariable (Table 2) regions of Msp2 in the St. Maries strain were represented by the design and synthesis of 30-mer, overlapping peptides (Fig. 1) [5], [6]. Sera from all animals obtained prior to immunization, 42 days after the last immunization, and 45 days after challenge with live organisms were tested. Enzyme-linked immunosorbent assays (ELISA) were done to map the anti-Msp2 antibody response. Immulon-II 96-well plates were coated with 1 μg of peptide per well in coating buffer (50 mM Na_2_CO_3_, pH 9.6) overnight at 4 °C, washed with PBS containing 0.05% (vol/vol) Tween20, and then blocked with PBS containing 5% (wt/vol) milk and 0.05% (vol/vol) Tween20 for 1 h. To determine the end-point titers, sera were diluted starting at 1:10 in blocking buffer. Dilutions were doubled until a signal was no longer detected or a dilution of 1:20,480 was reached. Titers were reported as the reciprocal of the last dilution in which antibody binding was detected. Fifty μl of each diluted serum sample were added to each well in triplicate. Following washing, 50 μl of 1:500 dilution of recombinant protein G-horseradish peroxidase (Zymed, Carlsbad, CA), to detect IgG binding, were added per well, and the plates incubated for 1 h at room temperature. After additional washes, binding of protein G to IgG was detected using Sureblue microwell peroxidase substrate (Kirkegaard and Perry Laboratories, Gaithersburg, MD) at 100 μl/well for 15 min. and stopped with 100 μl of 1% hydrochloric acid. The optical density at 450 nm was determined. Positive binding was statistically defined as exceeding the mean plus 2 standard deviations of the OD_450_ of preimmune serum from the same animal for the specific peptide, exceeding 2 times the absolute mean value of OD_450_ of the test serum with negative control peptide P1, and greater than the mean plus 2 standard deviations of the OD_450_ for a specific peptide from all control animals that received only adjuvant.

### 2.4 Determination of breadth and titer scores

To evaluate and compare the number of Msp2 epitopes recognized, breadth scores and mean titers were determined for each serum sample. To establish a breadth score, one point was given for each peptide recognized at a serum dilution of ≥1:10. All of the points for each conserved region peptide and all of the points for each HVR peptide were summed separately. In the order to compare the breadth scores between the CR and HVR peptides, the breadth score was divided by the number of peptides in each group. For example, animal 5933 had antibody recognizing 6 of the 15 CR peptides and 14 of the 18 HVR peptides, giving a CR breadth score of 0.40 and a HVR breadth score of 0.78 (Table 3). To establish a mean titer for a serum sample, the reciprocal of the end-point dilution for each peptide was summed and divided by the number of peptides recognized by that particular serum sample. The titer scores to the CR and HVR region peptides were determined separately. Correlations were determined by calculating the Spearman rank order correlation coefficient using SAS version 9.1.

## 3 Results

### 3.1 Does immunization shift the antibody response toward the conserved regions of Msp2?

**Fig. 2:**
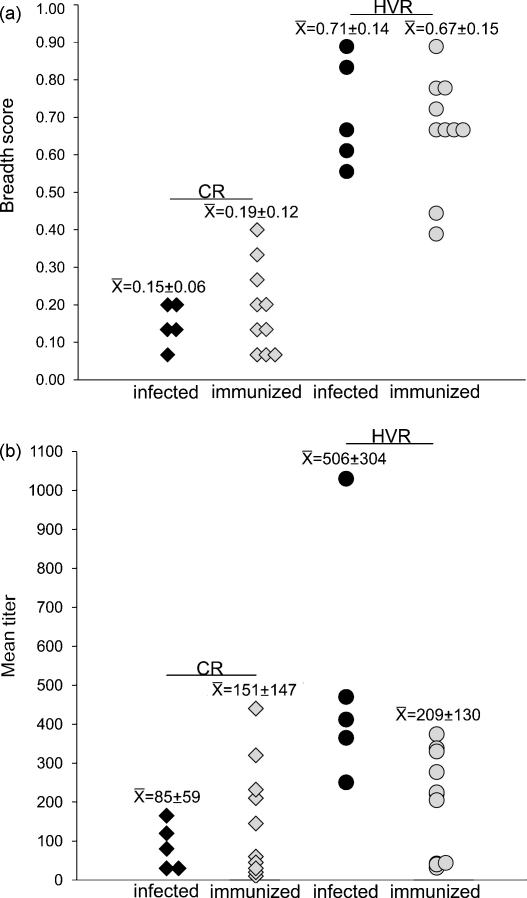
Comparison of the anti-Msp2 antibody response in animals infected with *A. marginale* and vaccinees. The antibody response specifically targeting the CR and HVR of Msp2 was determined using peptides representing each region of Msp2 in ELISAs. (a) The breadth score represents the mean number of peptides recognized by each animal at a ≥1:10 serum dilution. The mean ± SD for each group is reported above each column. (b) The mean titer to the CR and HVR for each serum sample was determined by summing the reciprocal dilutions of all end-point titers and dividing by the total number of peptides recognized at a ≥1:10 serum dilution. The mean ± SD for each group is reported above each column.

To address this question, the breadth and magnitude of the antibody response to all regions of Msp2 were compared in immunized animals and non-immunized, infected animals at the time of control of the initial bacteremia. Regardless of the treatment, the breadth scores to the HVR peptides were higher than the CR peptides (Fig. 2a). For example, the immunized animals had a mean breadth score of 0.19 ± 0.12 for the CR peptides and a score of 0.67 ± 0.15 for the HVR peptides; while the infected animals had a breadth score of 0.15 ± 0.06 for the CR peptides and 0.71 ± 0.14 for the HVR peptides.

The breadth scores to the CR peptides were slightly higher in the immunized animals (0.19 ± 0.12) than in the infected animals (0.15 ± 0.06). However, these differences were not statistically significant and are unlikely to be biologically relevant, as they predominantly represent differences between individual animals, and are due to the recognition of three additional CR peptides, P3, P15, and P14. P3 and P15 were recognized by vaccinee 5933. Although this animal had the highest breadth score (0.40) for the CR peptides, it also had the second highest bacteremia (4.5% infected erythrocytes) of the immunized animals (Table 3). P14 was solely recognized by vaccinee 5952. The breadth scores to the HVR peptides were similar when comparing the immunized and infected animals, with the scores in the infected animals marginally higher (Fig. 2a). When comparing titers, the immunized animals had higher titers to the CR of Msp2 than did the infected animals (Fig. 2b). However, the difference was not statistically significant and was attributed to the variation among individual animals. The infected cattle had higher titers to the HVR than did the vaccinees, however, this was primarily attributed an animal (5967) with markedly high titers. Similarly, there were no significant differences between the immunized and infected animals when evaluating the titers to individual peptides (Supplemental Fig. 1). Due to the wide variation among individuals within a group, we posed the following question: within a treatment group, is there a correlation between the control of bacteremia and the breadth or magnitude of the anti-Msp2 antibody response?

### 3.2 Does either the breadth or magnitude of the anti-Msp2 antibody response correlate with the control of bacteremia in infected animals?

**Fig. 3:**
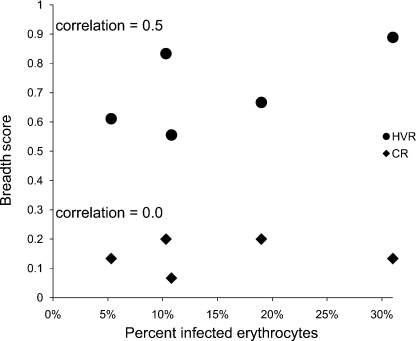
Comparison between the breadth of the anti-Msp2 antibody response and bacteremia in *A. marginale* infection. Bacteremia, as represented by the percent of infected erythrocytes, was determined by daily counting of *A. marginale* inclusion bodies in Giemsa stained blood smears. The antibody response specifically targeting the CR and HVR of Msp2 was determined using peptides representing each region of Msp2 in ELISAs. The breadth score represents the mean number of peptides recognized by each animal at a ≥1:10 serum dilution. Spearman rank order correlation coefficient is reported.

**Fig. 4:**
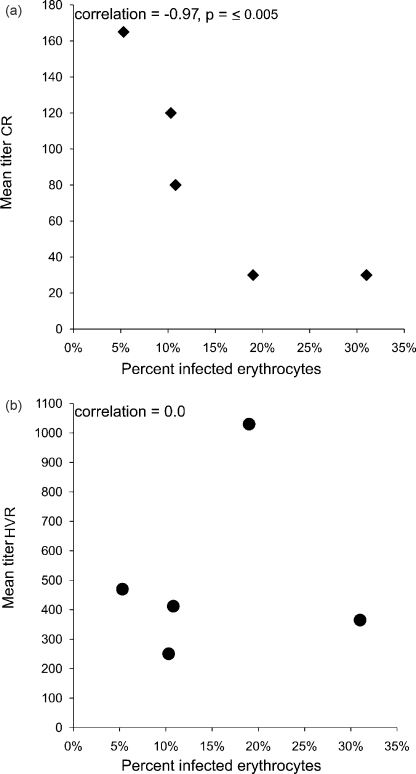
Comparison between the magnitude of the anti-Msp2 antibody response to the CR (a) and HVR (b) and bacteremia in *A. marginale* infection. Bacteremia, as represented by the percent of infected erythrocytes, was determined by daily counting of *A. marginale* inclusion bodies in Giemsa stained blood smears. The mean titers were determined by summing the reciprocal of the end-point dilution for each peptide recognized by a serum sample and dividing by the number of peptides recognized at ≥1:10 dilution. Spearman rank order correlation coefficient is reported.

Among the animals that were infected, there was no correlation between the breadth scores to either the CR or HVR peptides and bacteremia (Fig. 3). For example, one of the animals (5969) with the highest total breadth (including both the HVR and CR) score also had the highest bacteremia (31%). In contrast, there was a strong inverse correlation between bacteremia and titers to the CR (Fig. 4a), but not the HVR (Fig. 4b), of Msp2. Those animals with higher titers to the CR had lower levels of bacteremia (Spearman rank correlation coefficient = −0.97, *p* ≤ 0.005).

### 3.3 Does either the breadth or magnitude of the anti-Msp2 antibody response correlate with protection from infection in immunized animals?

**Fig. 5:**
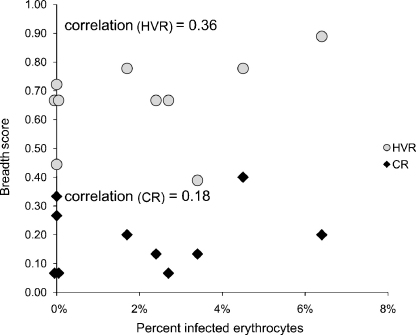
Comparison between the breadth of the antibody response to Msp2 and bacteremia following challenge in vaccinees. Bacteremia, as represented by the percent of infected erythrocytes, was determined by daily counting of *A. marginale* inclusion bodies in Giemsa stained blood smears. The antibody response specifically targeting the CR and HVR of Msp2 was determined using peptides representing each region of Msp2 in ELISAs. The breadth score represents the mean number of peptides recognized by each animal at a ≥1:10 serum dilution. Spearman rank order correlation coefficient is reported.

To address this question, only the immunized animals were considered. Because IgG2 levels have been associated with protection in immunized animals, both total IgG and IgG2 anti-Msp2 specific antibody to each region of Msp2 were measured [10]. The animals that did not develop infection (protection from infection) were compared to those that developed bacteremia. Among the immunized animals, when measuring total IgG, the breadth scores to CR and HVR peptides were similar when comparing the animals that were protected from infection to those that developed bacteremia (Fig. 5). For example, two of the animals with the lowest breadth score (0.07) to the CR peptides were protected from infection. Additionally, there were also no differences when comparing the total breadth score, which included the combined total IgG response to the both the CR and the HVR of Msp2. Findings were similar when measuring IgG2 (Supplemental Fig. 2). Two of the animals with the lowest breadth scores to the CR (<0.1) were protected from infection. The breadth scores to the HVR were higher, but again, there was no correlation between protection from infection and the breadth of the IgG2 specific responses to the HVR.

**Fig. 6:**
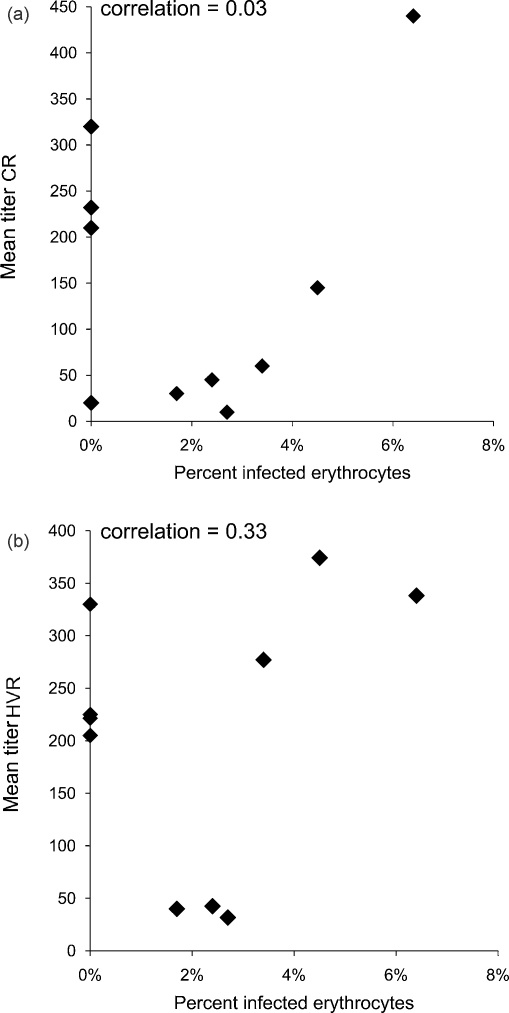
Comparison between the magnitude of the anti-Msp2 antibody response to the CR (a) and HVR (b) and bacteremia in vaccinees following challenge. Bacteremia, as represented by the percent of infected erythrocytes, was determined by daily counting of *A. marginale* inclusion bodies in Giemsa stained blood smears. The mean titers were determined by summing the reciprocal of the end-point dilution for each peptide recognized by a serum sample and dividing by the number of peptides recognized at ≥1:10 dilution. Spearman rank order correlation coefficient is reported.

There was no correlation between the titers to the CR and protection from infection when considering either total IgG or IgG2 only (Fig. 6a and Supplemental Fig. 3a). Three of the four animals that were protected from infection had total IgG CR titer scores above 200, while the remaining animal had a score of 20. The IgG2 titers scores to the CR varied from 0 to 160, while the range of scores in animals protected from infection varied from 18 to 160 (Supplemental Fig. 3a). Similarly, there was no correlation between protection from infection and titers to the HVR of Msp2 when considering either total IgG or IgG2 (Fig. 6b and Supplemental Fig. 3b). However, unlike the highly variable response to the CR, animals that were protected from infection had mid-range to high total IgG titers to the HVR peptides (205–330). Vaccinees that developed relatively high levels of bacteremia also had titers in this range. Among the animals that developed bacteremia, there was a trend toward vaccinees with high total IgG titers also having higher bacteremia.

## 4 Discussion

All groups of animals, including those that were infected, those that were immunized and protected from high-level bacteremia, and those that were immunized and completely protected from infection had similar anti-Msp2 antibody responses, in terms of both breadth and magnitude. Thus, we reject the hypothesis that immunization alters the anti-Msp2 antibody response as compared to infection. It is possible that there are variant Msp2 epitopes that we did not assess in these experiments, e.g. highly conformation-dependent epitopes not represented by the overlapping peptides or epitopes formed by the junction of two recombined oligopeptide segments. However, the length of peptides used in the assays, 30 amino acids, is relevant as this length represents the mean oligopeptide length encoded by segments recombined into the expression site during infection (29 ± 13 amino acids) [14]. Furthermore, Msp2 variable-region epitopes have been shown to be surface exposed, and development of variant-specific antibody is associated with variant clearance during persistent infection [8], [12], [15]. In addition to the lack of a shift in the breadth or magnitude of the anti-Msp2 antibody production in response to immunization, targeting of specific CR or HVR epitopes did not correlate with protective immunity. One possible exception was the conserved region epitope P5, which was recognized by four of ten of the immunized animals, two of which were protected from infection. None of the infected animals had antibody to P5.

Among the infected animals, the anti-Msp2 antibody response was measured during the control of the initial bacteremic peak. At this time point, high titers to the CR but not the HVR of Msp2 correlated with control of bacteremia. This was not the case in immunized animals in which there was no correlation between the anti-Msp2 antibody response and bacteremia, supporting the hypothesis that separate immunologic mechanisms control bacteremia in infected animals as compared to immunized animals. Among the immunized animals, there was no correlation between the breadth or magnitude of the anti-Msp2 antibody response and protection from infection. Among the animals that developed bacteremia in the face of immunization, there was a trend toward the animals with the highest titers to both the HVR and the CR also having the highest bacteremia. This was particularly true for the response to the HVR peptides I.1, III.1, and III.3. The reason for this is unknown, but helps to emphasize the point, that while immunized animals were better able to control bacteremia as compared to infected animals, epitopes other than those on Msp2 were likely responsible for that immunologic control.

A strong antibody response is known to be directed against Msp2 during acute infection. However, the data presented here fail to show a relationship between antibody to the HVR and control of bacteremia in either immunized or infected animals during acute infection. This was not due to a lack of Msp2 epitopes in the immunogen as immunization resulted in the production of antibody to all possible regions of Msp2, except one, thus we can infer that the immunogen contained a wide variety of the Msp2 epitopes. Additionally, the antibody repertoire did not change significantly in response to infection. Thus, a similar antigenic repertoire was available in the immunogen and during infection. These data suggest that the anti-Msp2 antibody response may be irrelevant during the control of the initial bacteremia, and are consistent with previously reported findings indicating that animals immunized with Msp2 variants were not protected when challenged with *A. marginale* expressing similar variants as those in the immunogen [16].

A theme among antigenically variant organisms is the ability to establish persistent infection which is generally clinically silent and characterized by low numbers of detectable organisms. Importantly during persistent infection, the adaptive immune response is able to control, but not clear infection. The inability to clear the infection is thought to be due to the generation of antigenically variant surface proteins which escape detection and allow for a window of pathogen replication [17]. For example, repeated exposure to *Plasmodium falciparum*, one of the causative agents of malaria, results in the development of naturally acquired immunity. In both *A. marginale* and *P. falciparum*, control of persistent infection is thought to be due in part to antibody directed toward surface expressed variant antigens. In the case of *A. marginale*, a temporal relationship exists between clearance of an Msp2 variant and development of a variant-specific antibody response [8], [9]. Similarly, *P. falciparum* parasites causing clinical disease express a PfEMP1 protein to which the patient has no pre-existing antibody; in response the immune system mounts an antibody response with specificity for the expressed protein [18], [19], [20], [21], [22]. Thus, it has been suggested that naturally acquired immunity to *P. falciparum* correlates with gradual acquisition of an entire repertoire of protective PfEMP1 antibody characterized by asymptomatic parasitemia, but does not result in sterile immunity or protection against re-infection, and requires years to develop [22], [23]. In contrast to naturally acquired immunity, sterile immunity can be induced by immunization with irradiated sporozoites in the case of *P. falciparum*, and outer membrane proteins, in the case of *A. marginale* [7], [10], [11], [24].

The data presented in this paper indicate that there is no correlation between the prevention of infection due to immunization and the antibody response to the highly immunogenic hypervariable surface protein responsible for immune evasion. Thus, the difference between the evasion of immunity resulting in persistent infection and the immunization-induced complete clearance is likely due to induction of antibody to conserved proteins that occurs following immunization but does not occur during natural infection. Although antibody to Msp2 is abundantly produced in response to immunization, antibodies targeting a wide variety of conserved proteins have also been identified [25]. Thus, shifting the immune response toward conserved epitopes that are poorly recognized during infection may be the key to effective vaccine development.

## Figures and Tables

**Table 1:** Amino acid sequence of the peptides representing the conserved expression site domains of Msp2.

Locus	Peptides	Amino acid sequence
Expression site N-terminal domain	P1	MSAVSNRKLPLGGVLMALVAAVAPIHSLLA
P2	AVAPIHSLLAAPAAGAGAGGEGLFSGAGAG
P3	EGLFSGAGAGSFYIGLDYSPAFGSIKDFKV
P4	AFGSIKDFKVQEAGGTTRGVFPYKRDAAGR
P5	FPYKRDAAGRVDFKVHNFDWSAPEPKISFK
P6	SAPEPKISFKDSMLTALEGSIGYSIGGARV
P7	IGYSIGGARVEVEVGYERFVIKGGKKSNED
P8	IKGGKKSNEDTASVFLLGKELAYDTARGQV
P9	LAYDTARGQVDRLAAALGKMTKGEAKRWG

Expression site C-terminal domain	P10	VAGAFARAVEGAEVIEVRAIGSTSVMLNAC
P11	GSTSVMLNACYDLLTDGIGVVPYACAGIGG
P12	VPYACAGIGGNFVSVVDGHINPKFAYRVKA
P13	NPKFAYRVKAGLSYALTPEISAFAGAFYHK
P14	SAFAGAFYHKVLGDGDYDELPLSPISDYTG
P15	PLSPISDYTGTAGKNKDTGIASFNFAYFGG
P16	TAGKNKDTGIASFNFAYFGGELGVRFAF

**Table 2:** Pseudogene designations of amino acid sequence of oligopeptides representing the complete HVR repertoire of Msp2.

Pseudogene donor locus	Peptides	Amino acid sequence
1	I.1^a^	TKSEAKKWGNAIESATGTTSGDELSKKVCG
	I.2	GDELSKKVCGKGTTSGNQCGVNATSGSTNN
	I.3	VNATSGSTNNGKLSTVFNTDGAEAISSMDT
	I.4	GAEAISSMDTTASGTSNTISLQGMAGNINS
2	I.1^a^	TKSEAKKWGNAIESATGTTSGDELSKKVCG
	II.2	GDELSKKVCGKGEGSNGTKKCGTTDSTATT
	II.3	CGTTDSTATTKISEVFTEGTDTLLSVEGNK
	II.4	DTLLSVEGNKDTINLQGMANNINNLSKEDK
P1	III.1	TKGEAKKWGNAVENATNGDKVSQNVCKGTG
	III.2	VSQNVCKGTGSTGSSGNKCGTTDSTATTKI
	III.3	TTDSTATTKISAVFTEDAAAQLSTMDNTTI
E6/F7	IV.1	TKGEAKKWGTTVEAATNGQTVSQKVCGNGT
	IV.2	VSQKVCGNGTGSSGSNCGKNTTDSTNNNGK
	IV.3	TTDSTNNNGKITQAFTADSDTTLLSAESSN
	IV.4	TTLLSAESSNISTSGMATNINGLSKEEKAV
9H1	V.1	TKSEAKKWGNAIESATGTTNGEKVSQKVCG
	V.2	GEKVSQKVCGNGTGSSGTQCGKNSGDTNGS
	V.3	GKNSGDTNGSSTTQHKISAVFTDEATLLSA
	V.4	FTDEATLLSAAGDTINTTGMAGNINSLTKD

a Pseudogene 1 and pseudogene 2 encode the identical N-terminal 30 amino acid HVR oligopeptide segment.

**Table 3:** The number of peptides and mean titers of the total IgG anti-Msp2 specific antibody response after immunization and after challenge.

Immunogen	Animal ID	Immunized^a^				Bacteremia
		CR		HVR		
		# peptides recognized^b^	Mean (Max) titer^c^	# peptides recognized	Mean (Max) titer	Max. % infected RBCs
Complexes	5933	6	145 (640)	14	374 (1280)	4.5%
Complexes	5961	2	60 (640)	7	277 (1280)	3.4%
Complexes	5972	1	10 (10)	12	32 (80)	2.7%
Complexes	5946	4	210 (640)	13	222 (640)	Protected^d^
Complexes	5952	5	232 (640)	12	225 (640)	Protected

	Mean	2.8	131	11.6	226	

Outer membranes	5953	3	440 (640)	16	338 (1280)	6.4%
Outer membranes	5966	2	45 (80)	12	43 (160)	2.4%
Outer membranes	5978	3	30 (40)	14	40 (40)	1.7%
Outer membranes	5982	1	320 (320)	12	205 (320)	Protected
Outer membranes	5975	1	20 (20)	8	330 (640)	Protected

	Mean	2.0	171	12.4	191	

Infected^e^						
Adjuvant	5969	2	30 (40)	16	365 (1280)	31.0%
Adjuvant	5967	3	30 (40)	12	1030 (5120)	19.0%
Adjuvant	5958	1	80 (80)	10	412 (1280)	10.8%
Adjuvant	5979	3	120 (320)	15	251 (1280)	10.3%
Adjuvant	5974	2	165 (320)	11	470 (1280)	5.3%

	Mean	2.2	85	12.8	506	

a Specific antibody response measured after the final immunization and prior to challenge.

b The number of peptides recognized from either the CR or HVR at a 1:10 serum dilution.

c The mean of the titers for all peptides recognized by each serum sample. The maximum titer for each serum sample is reported in parentheses.

d Protected from infection as defined by negative PCR and repeated negative blood smears.

e Specific antibody response measured 45 days after infection with *A. marginale*.

## Appendix A Supplementary data

Supplementary Fig. 1 The magnitude of the anti-Msp2 antibody response to the CR (a) and HVR (b) to each peptide in animals infected with *A. marginale* and vaccinees. The mean titers were determined by summing the reciprocal of the end-point dilution for each individual peptide and dividing by the number of animals that recognized that peptide at ≥1:10 dilution. A single diamond on the *X*-axis represents all animals within a group that had no response to a particular peptide.

Supplementary Fig. 2 Comparison between the breadth of the IgG2 antibody response to Msp2 and bacteremia in vaccinees following challenge. Bacteremia, as represented by the percent of infected erythrocytes, was determined by daily counting of *A. marginale* inclusion bodies in Giemsa stained blood smears. The IgG2 antibody response specifically targeting the CR and HVR of Msp2 was determined using peptides representing each region of Msp2 in ELISAs. The breadth score represents the mean number of peptides recognized by each animal at a ≥1:10 serum dilution. Spearman rank order correlation coefficient is reported.

Supplementary Fig. 3 Comparison between the magnitude of the IgG2 anti-Msp2 antibody response to the CR (a) and HVR (b) and bacteremia in vaccinees following challenge. Bacteremia, as represented by the percent of infected erythrocytes, was determined by daily counting of *A. marginale* inclusion bodies in Giemsa stained blood smears. The mean titers were determined by summing the reciprocal of the end-point dilution for each peptide recognized by a serum sample and dividing by the number of peptides recognized at ≥1:10 dilution. Spearman rank order correlation coefficient is reported.
