# Supplementary material for: The immunization-induced antibody response to the Anaplasma marginale major surface protein 2 and its association with protective immunity
Source: Vaccine. 2010 May 7;28(21):3741–7. doi: 10.1016/j.vaccine.2010.02.067 (PMC2877794; doi:10.1016/j.vaccine.2010.02.067)
Supplement: Supplementary Fig. 1 — The magnitude of the anti-Msp2 antibody response to the CR (a) and HVR (b) to each peptide in animals infected with A. marginale and vaccinees. The mean titers were determined by summing the reciprocal of the end-point dilution for each individual peptide and dividing by the number of animals that recognized that peptide at ≥1:10 dilution. A single diamond on the X-axis represents all animals within a group that had no response to a particular peptide. [file mmc1.doc]

Supplemental Fig. 1. The magnitude of the anti-Msp2 antibody response to the CR (a) and the HVR (b) to each peptide in animals infected with *A. marginale* and vaccinees. The mean titers were determined by summing the reciprocal of the end-point dilution for each individual peptide and dividing by the number of animals that recognized that peptide at ≥ 1:10 dilution. A single diamond on the X-axis represents all animals within a group that had no response to a particular peptide.
