# Supplementary material for: The immunization-induced antibody response to the Anaplasma marginale major surface protein 2 and its association with protective immunity
Source: Vaccine. 2010 May 7;28(21):3741–7. doi: 10.1016/j.vaccine.2010.02.067 (PMC2877794; doi:10.1016/j.vaccine.2010.02.067)
Supplement: Supplementary Fig. 2 — Comparison between the breadth of the IgG2 antibody response to Msp2 and bacteremia in vaccinees following challenge. Bacteremia, as represented by the percent of infected erythrocytes, was determined by daily counting of A. marginale inclusion bodies in Giemsa stained blood smears. The IgG2 antibody response specifically targeting the CR and HVR of Msp2 was determined using peptides representing each region of Msp2 in ELISAs. The breadth score represents the mean number of peptides recognized by each animal at a ≥1:10 serum dilution. Spearman rank order correlation coefficient is reported. [file mmc2.doc]

Supplemental Fig. 2 Comparison between the breadth of the IgG2 antibody response to Msp2 and bacteremia in vaccinees following challenge. Bacteremia, as represented by the percent of infected erythrocytes, was determined by daily counting of *A. marginale* inclusion bodies in Giemsa stained blood smears. The IgG2 antibody response specifically targeting the CR and HVR of Msp2 was determined using peptides representing each region of Msp2 in ELISAs. The breadth score represents the mean number of peptides recognized by each animal at a ≥1:10 serum dilution. The Spearman rank order correlation coefficient is reported.
