# Supplementary material for: The immunization-induced antibody response to the Anaplasma marginale major surface protein 2 and its association with protective immunity
Source: Vaccine. 2010 May 7;28(21):3741–7. doi: 10.1016/j.vaccine.2010.02.067 (PMC2877794; doi:10.1016/j.vaccine.2010.02.067)
Supplement: Supplementary Fig. 3 — Comparison between the magnitude of the IgG2 anti-Msp2 antibody response to the CR (a) and HVR (b) and bacteremia in vaccinees following challenge. Bacteremia, as represented by the percent of infected erythrocytes, was determined by daily counting of A. marginale inclusion bodies in Giemsa stained blood smears. The mean titers were determined by summing the reciprocal of the end-point dilution for each peptide recognized by a serum sample and dividing by the number of peptides recognized at ≥1:10 dilution. Spearman rank order correlation coefficient is reported. [file mmc3.doc]

Supplemental Fig. 3. Comparison between the magnitude of the IgG2 anti-Msp2 antibody response to the CR (a) and the HVR (b) and bacteremia in vaccinees following challenge. Bacteremia, as represented by the percent of infected erythrocytes, was determined by daily counting of *A. marginale* inclusion bodies in Giemsa stained blood smears. The mean titers were determined by summing the reciprocal of the end-point dilution for each peptide recognized by a serum sample and dividing by the number of peptides recognized at ≥ 1:10 dilution. The Spearman rank order correlation coefficient is reported.
